# Bulk RNA Sequencing With Integrated Single-Cell RNA Sequencing Identifies BCL2A1 as a Potential Diagnostic and Prognostic Biomarker for Sepsis

**DOI:** 10.3389/fpubh.2022.937303

**Published:** 2022-06-27

**Authors:** Jun Li, Mi Zhou, Jia-Qi Feng, Soon-Min Hong, Shao-Ying Yang, Lang-Xian Zhi, Wan-Yi Lin, Cheng Zhu, Yue-Tian Yu, Liang-Jing Lu

**Affiliations:** ^1^Department of Rheumatology, Renji Hospital, Shanghai Jiaotong University School of Medicine, Shanghai, China; ^2^Department of Disease Prevention and Control, Ruijin Hospital, Shanghai Jiao Tong University School of Medicine, Shanghai, China; ^3^Department of Critical Care Medicine, Renji Hospital, Shanghai Jiao Tong University School of Medicine, Shanghai, China

**Keywords:** sepsis, diagnosis, biomarker, single-cell, sequencing

## Abstract

**Background:**

Sepsis is one of the leading causes of morbidity and mortality worldwide in the intensive care unit (ICU). The prognosis of the disease strongly depends on rapid diagnosis and appropriate treatment. Thus, some new and accurate sepsis-related biomarkers are pressing needed and their efficiency should be carefully demonstrated.

**Methods:**

Differential expression analysis and weighted gene co-expression network analysis (WGCNA) were applied to detect sepsis and monocyte/macrophage-related genes. Least absolute shrinkage and selection operator (LASSO) and random forest regression analyses were used in combination to screen out prognostic genes. Single-cell RNA sequence profiling was utilized to further verify the expression of these genes on a single cell level. Receiver operating characteristic (ROC) curve and decision curve analysis (DCA) were also applied to verify the diagnostic value of the target biomarkers.

**Results:**

The intersections of the genes detected by differential expression and WGCNA analyses identified 141 overlapping candidate genes that were closely related to sepsis and macrophages. The LASSO and random forest regression analyses further screened out 17 prognostic genes. Single-cell RNA sequencing analysis detected that *FCGR1A* and *BCL2A1* might be potential biomarkers for sepsis diagnosis and the diagnostic efficacy of *BCL2A1* was further validated by ROC curve and DCA.

**Conclusions:**

It was revealed that *BCL2A1* had good diagnostic and prognostic value for sepsis, and that it can be applied as a potential and novel biomarker for the management of the disease.

## Introduction

Sepsis, which is defined as “life-threatening organ dysfunction caused by a dysregulated host response to infection,” is one of the leading causes of morbidity and mortality worldwide in the intensive care unit (ICU) (1). As a disease of heterogenous and imprecise syndromes, it also has multiple phenotypes. Based on some phenotypic identification methods of sepsis, most of the patients are grouped according to the degree of inflammatory response, the stability of hemodynamic, the severity of coagulopathy and by using the clinical or genomic variables (2).

Regardless of the subtypes and phenotypic of the disease, the prognosis of sepsis strongly depends on rapid diagnosis and appropriate treatment (1, 3). Therefore, it is particularly important to find accurate, sensitive, and early biomarkers for diagnosing sepsis. To date, multiple biomarkers have been identified and applied for the diagnosis of the disease, including several classic biomarkers [C-reactive protein (CRP) and procalcitonin (PCT)], and some novel ones (decoy receptor-3 and hepcidin) (4, 5). However, classical biomarkers such as CRP and PCT lack specificity, which makes diagnosing sepsis in its early stages extremely difficult. At the same time, the precise roles of newly identified biomarkers such as decoy receptor-3 and hepcidin in the management of patients with septic shock have not been well defined. Moreover, among the biomarkers that have been studied, only a few have been properly evaluated in large cohort studies (6).

Previous studies have shown that monocytes and macrophages play an important role in the pathogenesis of sepsis (7). Recent studies have also demonstrated that monocytes can facilitate the proliferation and exhaustion of T cells via interleukin-1B (IL-1B) signaling pathways and finally lead to monocyte-dependent suppression of T cell function in sepsis (7). Furthermore, monocytes can be activated through aggregation with platelets and release multiple proinflammatory cytokines [e.g., IL-6, IL-1β, and tumor necrosis factor-α (TNF-α)]. Platelet-monocyte aggregates can also facilitate polarization of CD14+ monocytes toward a proinflammatory M1 phenotype (8). Moreover, macrophage polarization has been found to be closely related to the pathogenesis of sepsis (9). Macrophages can mainly polarize into two distinct phenotypes with opposite influences on immune function: M1-like macrophages with proinflammatory function and M2-like macrophages with anti-inflammatory function. In patients with sepsis, elevated levels of multiple cytokines [TNF-α and interferon-γ (IFN-γ)] and pathogen-related molecular patterns [e.g., lipopolysaccharide (LPS)] can activate inflammatory pathways, especially the nuclear factor-κB (NF-κB) pathway, and eventually trigger macrophage polarization toward the M1 phenotype. A continuous M1-like macrophage polarization can further induce an inflammatory response and cause organ, tissue, and immune cell damage (9).

Considering the key roles of monocytes and macrophages in the pathogenesis of sepsis, our study aims to screen some novel diagnostic and prognostic biomarkers that are related to monocytes and macrophages by using bulk RNA sequencing with integrated single-cell RNA sequencing. We hypothesis that the identified novel sepsis-related biomarkers might provide new ideals and research directions for the diagnosis and treatment of sepsis.

## Methods

### Bulk RNA-Sequencing Data Downloading and Processing

The datasets GSE65682, GSE28750, GSE69528, and GSE100159 were downloaded from the Gene Expression Omnibus (GEO) database (https://www.ncbi.nlm.nih.gov/geo/) using the GEOquery R package (version 2.60.0, The R Foundation for Statistical Computing, Vienna, Austria) (10). We designated GSE65682 as the training set for the downstream analysis, and GSE28750, GSE69528, and GSE100159 were designated as the validation sets to confirm the results. The GSE65682 dataset included data on 760 ICU patients and 42 healthy cases. Differentially expressed genes (DEGs) were detected using the “limma” R package which had been recognized as a classic algorithm in bioinformatics analysis (11). The DEGs with an adjusted *P*-value (adj. P. val.) of <0.05 and |log FC| ≥1.5 were considered statistically significant.

### WGCNA Network Construction

Clusters of highly correlated genes were screened and a weighted gene co-expression network was constructed using the WGCNA algorithm which is a widely used approach to identify potential biomarkers of interest (12). The soft-threshold β was set to eight to ensure the network followed a scale-free distribution. Next, the adjacency matrix was transformed into a topological overlap matrix (TOM). Subsequently, hierarchical clustering was applied to generate modules and every module consisted of at least 30 genes (min Module Size = 30). Finally, the module eigengene (ME) was calculated, and cluster analysis was performed on the modules. Modules that were similar were merged into a new module.

### Identification of Clinically Significant and Immune Cell Infiltration-Related Module

We performed immune infiltration profiling on the samples of the GSE65682 dataset using the Cell-type Identification by Estimating Relative Subsets of RNA Transcripts (CIBERSORT) deconvolution algorithm (13). The algorithm can count the immune cell infiltration score and quantify the degree of infiltration of 22 types of immune cells on target samples. Next, gene significance (GS) and module membership (MM) were calculated to evaluate the association between the modules and sample traits (including immune cell infiltration score and clinical data). Finally, clinical significance and immune cell infiltration-related modules were identified, and hub genes of this module were extracted for subsequent analysis.

### LASSO and Random Forest Regression

LASSO and random forest regression has been widely applied to screen prognosis-related genes in the previous studies (14, 15). Thus, they were utilized in combination to identify the genes that were correlated with the prognosis of sepsis. The LASSO regression analysis was conducted using the “glmnet” R package, and random forest regression analysis was performed using the “randomForest” R package.

### ROC Curve and DCA Analysis

The receiver operating characteristic (ROC) curve analysis was performed using the “pROC” R package and was visualized using the “ggplot2” R package. The decision curve analysis (DCA) was conducted using the “DecisionCurve” R package.

### Single-Cell RNA Sequencing Data

Bacterial sepsis data were downloaded from the SCP548 of the Broad Institute Single Cell Portal (SCP) (https://singlecell.broadinstitute.org/single_cell). The single-cell data includes 19 healthy control samples from Research Blood Components (Watertown, MA, USA) and 46 infected samples from three different medical services (an emergency department, a medical department and an ICU) (16). As our study was focusing on patients with sepsis, we extracted the data from Bac-Sep (defined as having bacteremia and sepsis but not requiring ICU admission, *n* = 4), ICU-Sep (defined as patients with sepsis requiring ICU care, *n* = 8), and healthy controls (*n* = 19) for subsequent analysis. The data of coronavirus disease of 2019 (COVID-19) was also downloaded from the GSE150728 of the GEO database. This data was extracted from seven hospitalized patients due to COVID-19 and six healthy controls.

### Single-Cell RNA Sequencing Data Processing and Analysis

The bacterial sepsis datasets were based on the 10x Genomics platform (https://www.10xgenomics.com). We used the Seurat pipeline to analyze the single-cell RNA (scRNA) data. The original data matrix downloaded from SCP was inputted into R (version 4.1.1) and processed with the Seurat R package (version 4.0.4) (17). The “Create Seurat Object” function was utilized to transform the dataset into a “Seurat object.”

Quality control was conducted through filtering out cells with <200 genes, >2,500 genes, or >10% mitochondrial genes. A total of 60,543 filtered cells were included in the subsequent analysis. Data normalization was performed using the “LogNormalize” method, and 2,000 highly variable genes (HVGs) were identified using the “vst” method. Subsequently, the “Harmony” R package (version 0.1.0) was utilized to remove the batch effect of the sample identity (18). Cell cycle scores for every cell were calculated using the “CellCycleScoring” function, and the cell cycle effect was removed using the “Scaledata” function. Subsequently, we applied principal component analysis (PCA) to identify significant principal components (PCs) and to choose 30 PCs for t-distributed stochastic neighbor embedding (t-SNE) analysis. Moreover, 15 different clusters were identified using the “FindClusters” function with a parameter resolution of 0.6. Finally, we used a published list of marker genes to annotate the cell type of each cluster. The GSE150728 scRNA sequencing dataset was processed as described above. In all, 72,849 cells were included in the analysis. Cluster analysis was performed through the “FindNeighbors” and “FindClusters” functions at a resolution of 0.8.

### Immunity-Related Genes Score

The DEGs of each cluster between the control group and the disease group were screened using the “FindMarkers” function. Then, we used the ImmPort database (https://www.immport.org/shared/home) (19) to screen the DEGs of each cluster and identified immunity-related genes (IRGs). The IRGs were considered as a gene set to calculate the IRG scores in every cell using the AUCell R package (version 1.14.0) (20). The IRGs scores were calculated based on gene set enrichment analysis (GSEA). The cell which expresses more genes within the IRGs revealed a higher area under the curve (AUC) value. The threshold to distinguish gene set active cells was determined using the “AUCell explore Thresholds” function. Finally, we used the “ggplot2” R package (version 3.3.5) to visualize the active clusters by mapping the IRGs score in every cell relative to the t-SNE.

### Gene Ontology and/or GSEA

The Metascape website (https://metascape.org/gp/index.html) was used for functional enrichment analysis upon the hub genes of target module in bulk-sequencing profiling. The ClusterProfiler package (21) was utilized for performing Gene Ontology (GO) and GSEA on the marker genes of cell clusters in scRNA-sequencing profiling, where *p* < 0.05 indicated statistically significant enrichment.

## Results

**Figure 1:**
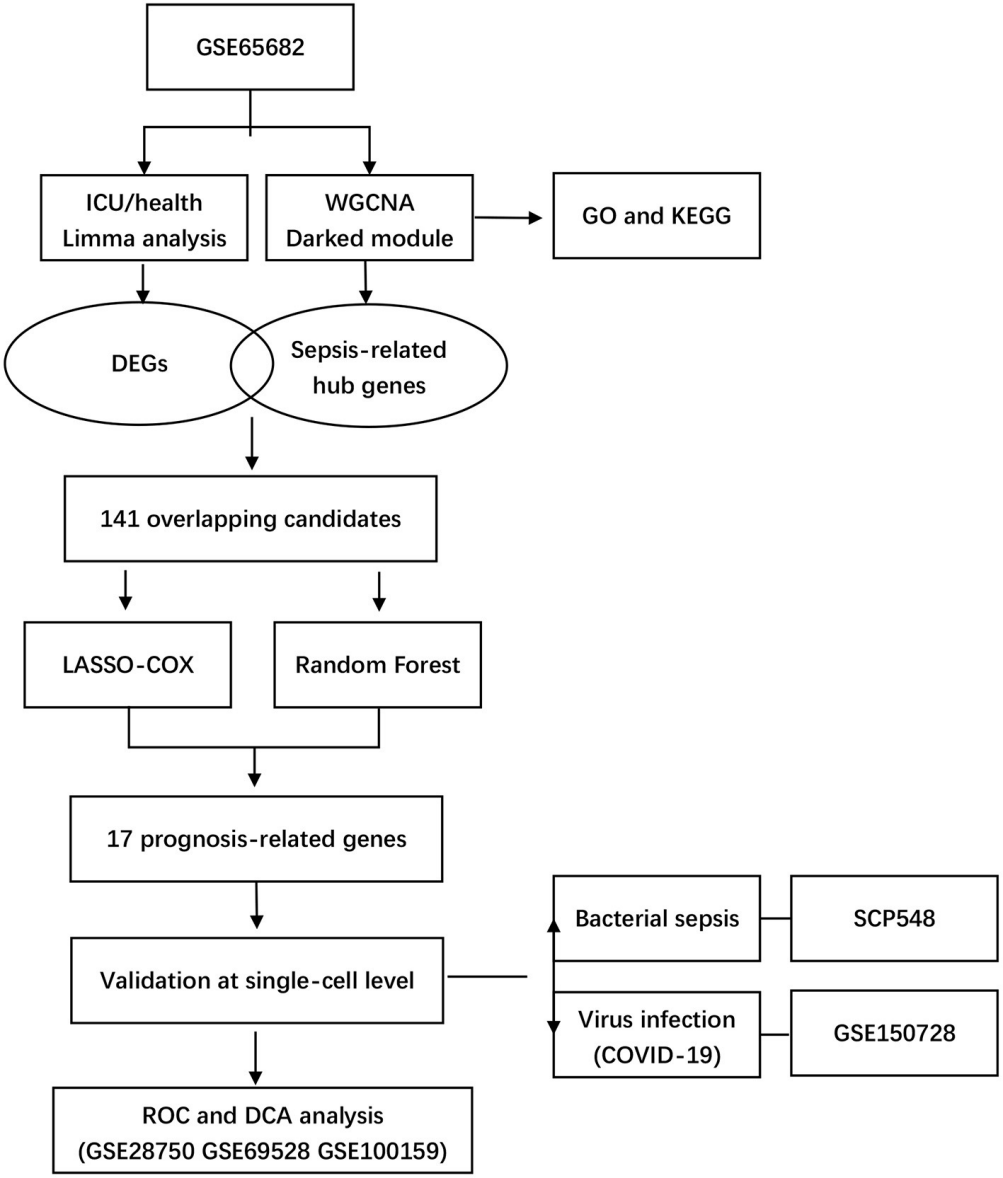
Flowchart of study.

Our study integrated four bulk-seq datasets and two scRNA-seq datasets. All the datasets included in our study were shown in detail in Table 1. The flowchart of our study was shown in Figure 1.

**Table 1:** Information for selected datasets in this study.

**Datasets**	**Type**	**Platform**	**Sample size (Disease/Control)**	**Cells (Disease/Control)**
GSE65682 (Training set)	Microarray	GPL13667	720/42 (ICU/health)	NA
SCP548 (Validation set)	scRNA sequencing	Illumina Novaseq S2 (Homo sapiens)	12/19 (sepsis/health)	13438/47105 (sepsis/health)
GSE150728 (Validation set)	scRNA sequencing	Illumina NovaSeq 6000 (Homo sapiens)	7/6 (COVID-19/health)	45105/27744 (COVID-19/health)
GSE28750 (Validation set)	Microarray	GPL570	10/20 (sepsis/health)	NA
GSE69528 (Validation set)	Microarray	GPL10558	83/55 (sepsis/health)	NA
GSE100159 (Validation set)	Microarray	GPL6884	35/11 (sepsis/health)	NA

### Identification of DEGs in the GSE65682 Dataset

**Figure 2:**
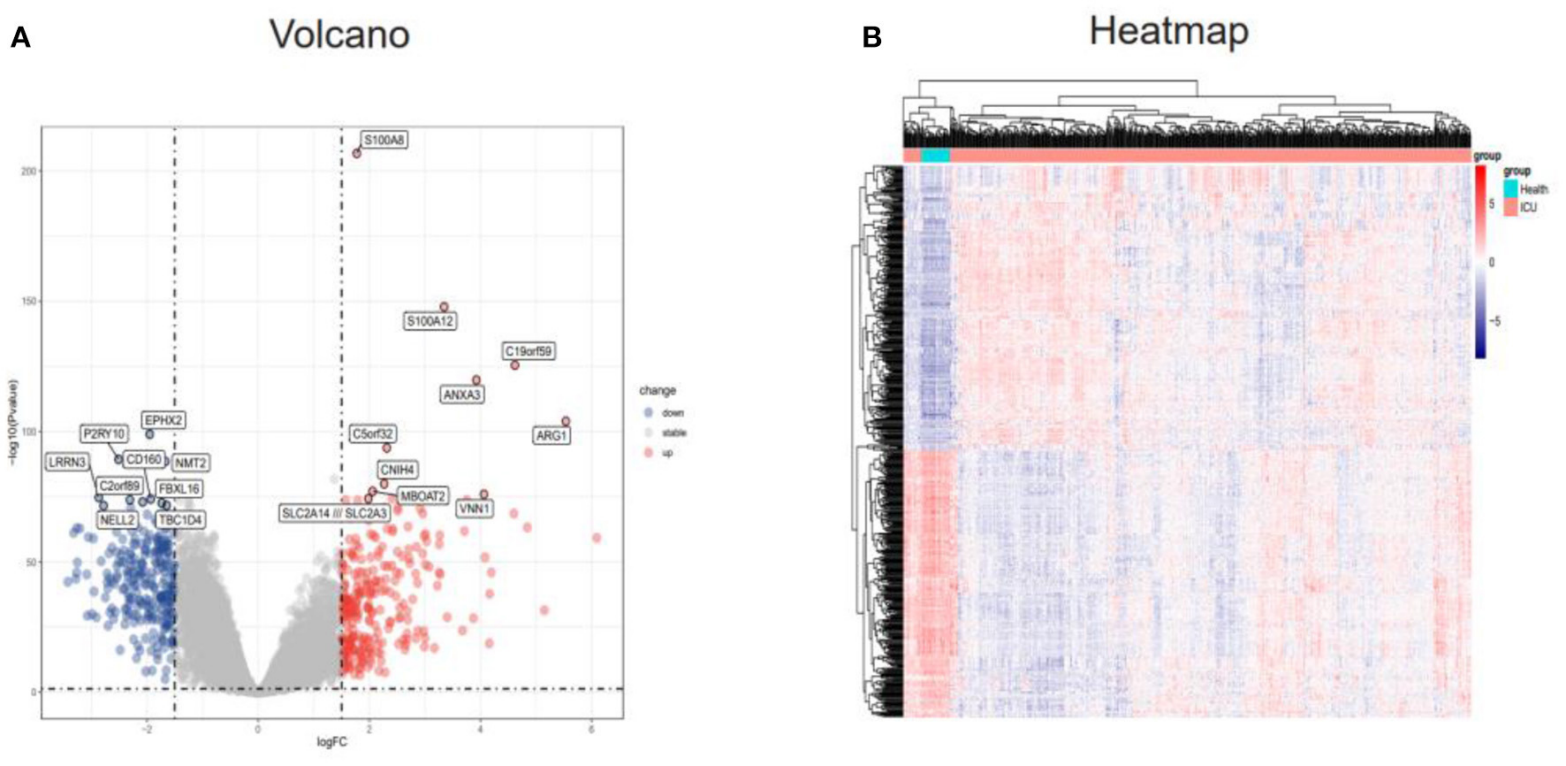
Identification of DEGs in the GSE65682 dataset. **(A)** Volcano plot of the DEGs (|logFC| > 1.5 and adjusted P-value l < 0.05) in GSE65682. Upregulated genes are in red, and downregulated genes are in blue. **(B)** Heatmap of the DEGs in GSE65682. DEGs, differentially expressed genes.

The “limma” R package was used to detect DEGs between the ICU samples and healthy samples of the GSE65682 dataset. The DEGs were screened according to an adj. P. val. of <0.05 and |log FC| ≥1.5. After screening the dataset, a total of 524 DEGs were obtained, of which 270 genes were upregulated and 254 genes were downregulated (Supplementary Table 1). These results were visualized using a volcano map (Figure 2A) and a heatmap (Figure 2B).

### Weighted Co-expression Network Construction and Identification of key Modules

**Figure 3:**
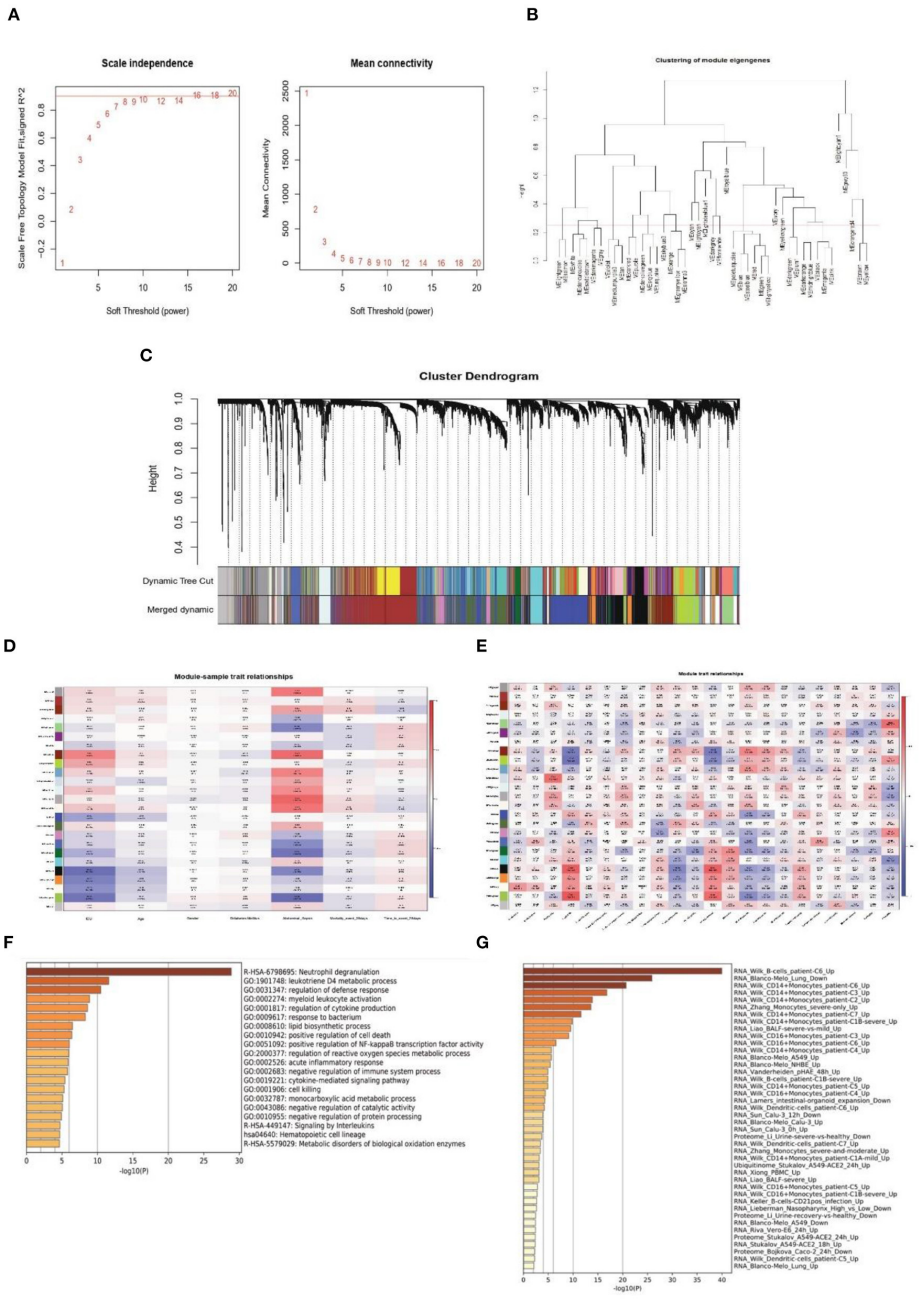
Weighted co-expression network construction and identification of key modules. **(A)** Correlation between the modules in the network topology analysis for various soft-thresholding powers. **(B)** Cut height for merging modules. **(C)** Gene dendrogram and related module colors. **(D)** Correlation between the 22 modules and sample traits. **(E)** Correlation between the 22 modules and immune cell scores. **(F,G)** Functional enrichment analysis of the Darked module genes.

Next, WGCNA analysis was conducted to detect the co-expression genes and modules based on 720 samples from the GSE65682 dataset. To ensure the network followed a scale-free distribution, a soft threshold power of eight was chosen as the most appropriate one for network construction (Figure 3A). Hierarchical clustering analysis was then performed to generate modules, and similar modules were merged. The cut height for merging modules was 0.25, which meant that modules whose eigengenes were correlated above 0.75 were merged (Figure 3B). Finally, 22 distinct gene co-expression modules were constructed, and these are shown in different colors in Figure 3C.

To identify the clinical significance and immune cell infiltration-related modules, the CIBERSORT deconvolution algorithm was used first to calculate the immune cell scores. Subsequently, GS and MM were calculated to evaluate the correlation between the modules and sample characteristics (including immune cell infiltration score and clinical data) (Figures 3D,E). Finally, the Darked module was chosen as the target modules. This module showed the strongest significant correlation with both admission to ICU (*r* = 0.46, *p* = 2e-43) and occurrence of abdominal sepsis (*r* = 0.57, *p* = 1e-69). Furthermore, the Darked module showed a positive association with macrophages M0 (*r* = 0.44, *p* = 1e-39) (Figures 3D,E). To further explore the function of the Darked module, Kyoto Encyclopedia of Genes and Genomes (KEGG) and GO enrichment analyses were performed on the hub genes of this module. It was apparent that the Darked module was mostly enriched in the inflammatory and infection-related pathway (Figure 3F). Furthermore, the function of the Darked module was also closely related to monocytes/macrophages, which is consistent with the results of the WGCNA analysis (Figure 3G). These results indicated that the patients with sepsis might show predominately a monocyte/macrophage infiltration.

### Identification of Prognosis-Related Genes

**Figure 4:**
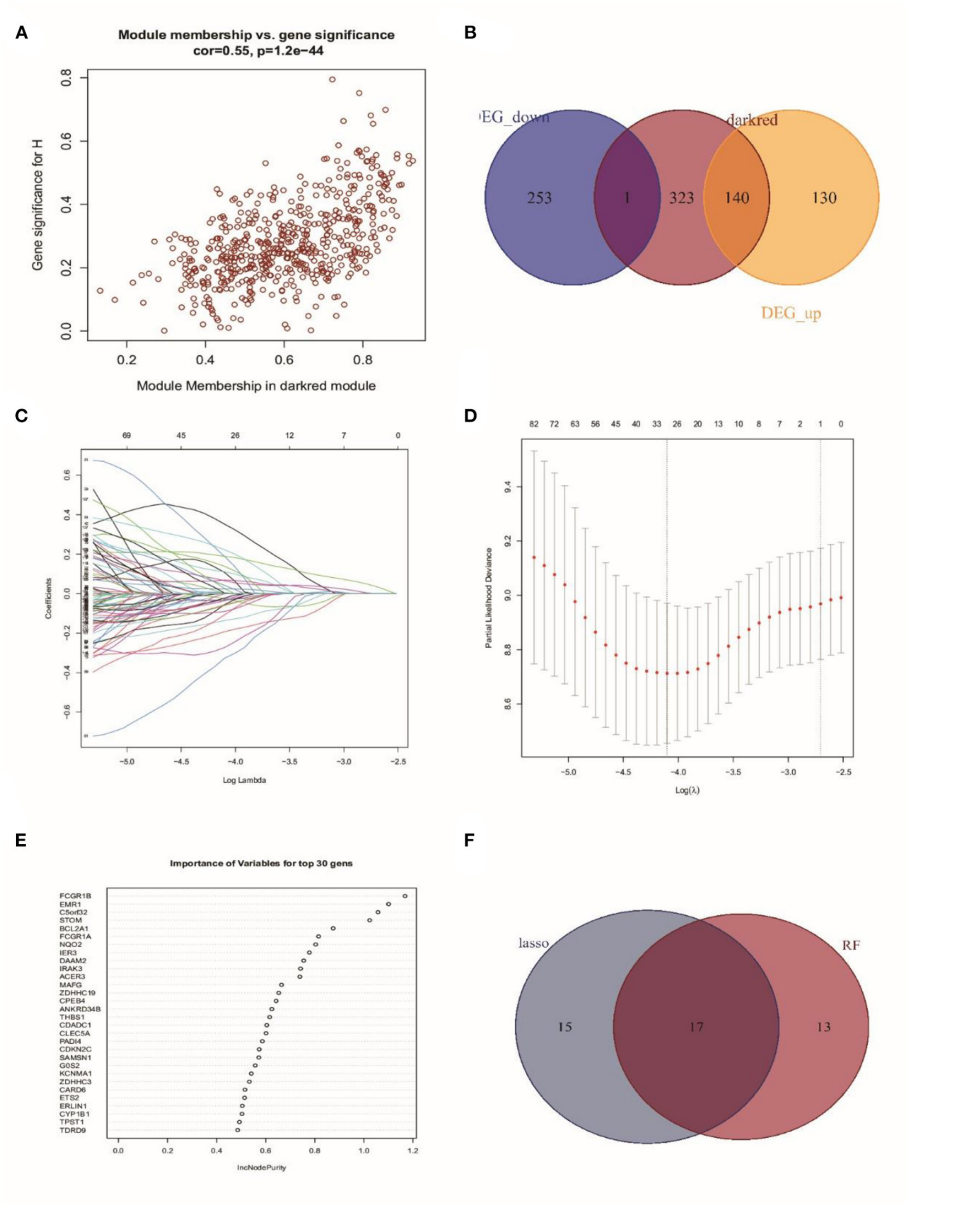
Identification of prognostic genes in patients with sepsis. **(A)** Extraction of the hub genes of the Darked module. **(B)** 141 overlapping candidates in the intersection of the DEGs and the Darked module genes. **(C,D)** LASSO Cox analysis identified 32 prognostic genes. **(E)** The top 30 prognostic genes in the random forest regression analysis. **(F)** Venn diagram showed the genes identified by LASSO Cox and random forest regression analyses. A total of 17 prognostic genes were found in the intersection of the results of from LASSO Cox and random forest regressions.

As the Darked module revealed a tight correlation with the status of sepsis, we extracted the hub genes of the Darked module for the following analysis (Figure 4A). The Darked module consisted of 463 genes. The intersection of hub genes among the Darked module and DEGs in the GSE65682 dataset were taken, and 141 genes were obtained, as shown in Figure 4B.

Subsequently, LASSO regression analysis in conjunction with random forest regression analysis was used to identify the prognostic genes (Figures 4C–E). Taking the intersections of the target genes screened out by the two algorithms, a Venn diagram was constructed (Figure 4F). A total of 32 genes in LASSO regression algorithms and 30 genes in random forest algorithms were retained. More importantly, 17 genes were obtained in both LASSO regression analysis and random forest analysis. The list of these 17 genes is shown in Supplementary Table 2.

### ScRNA Profiling of PBMCs in Bacterial Sepsis

**Figure 5:**
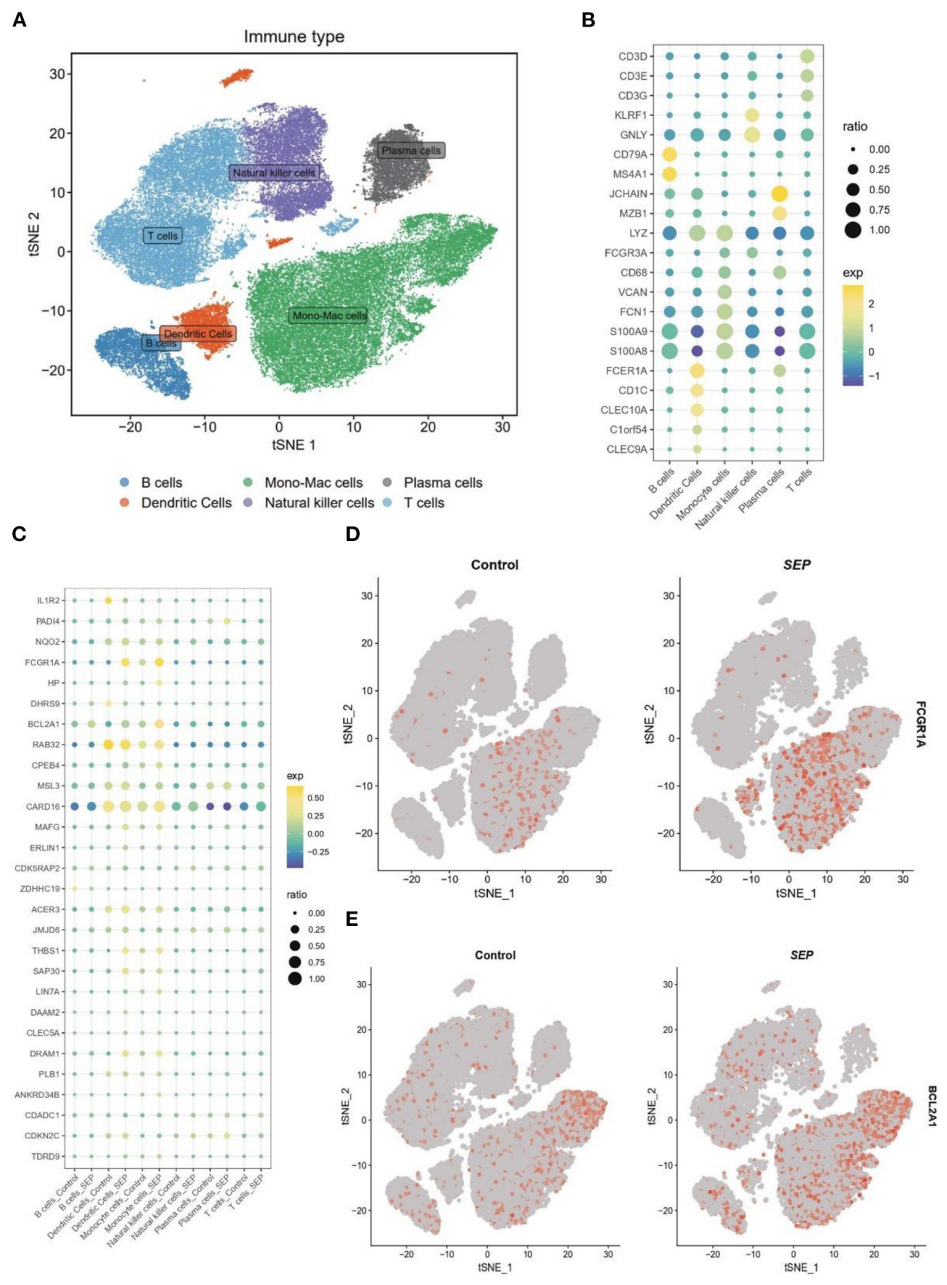
Differential expression of target genes on a single-cell level. **(A)** t-SNE plot of 60,543 cells from SCP548 datasets. Distinct cell types are shown in different colors. **(B)** Dot plot showing the respective marker genes of different cell types. **(C)** A dot plot showing that the expression levels of 17 genes differ between the sepsis and healthy samples. **(D,E)** Expression of *FCGR1A* and *BCL2A1* shown using a t-SNE plot. t-SNE, t-distributed stochastic neighbor embedding.

To confirm the result of bulk-RNA sequencing profiling, bacterial scRNA-sequencing data were analyzed. After data processing, 60,543 cells comprising 13,438 cells from patients with sepsis and 47,105 cells from healthy controls were retained. Subsequently, 15 clusters were identified via the t-SNE analysis of unsupervised clustering. These clusters were then annotated into six cell types based on the marker genes reported in the previous study. The six cell types were visualized using t-SNE analysis (Figure 5A). The expression of cell type marker genes was shown in a dot plot (Figure 5B).

Next, the expression level of the 17 genes, which had been screened out by the bulk RNA-sequencing profiling, was then examined at the single-cell level (Figure 5C). Finally, *FCGR1A* and *BCL2A1* were found to be considerably increased in the sepsis group. More importantly, both of them were predominantly expressed in the monocyte/macrophage cluster, which indicated that the two genes might be closely related to the function of this cluster (Figures 5D,E).

**Figure 6:**
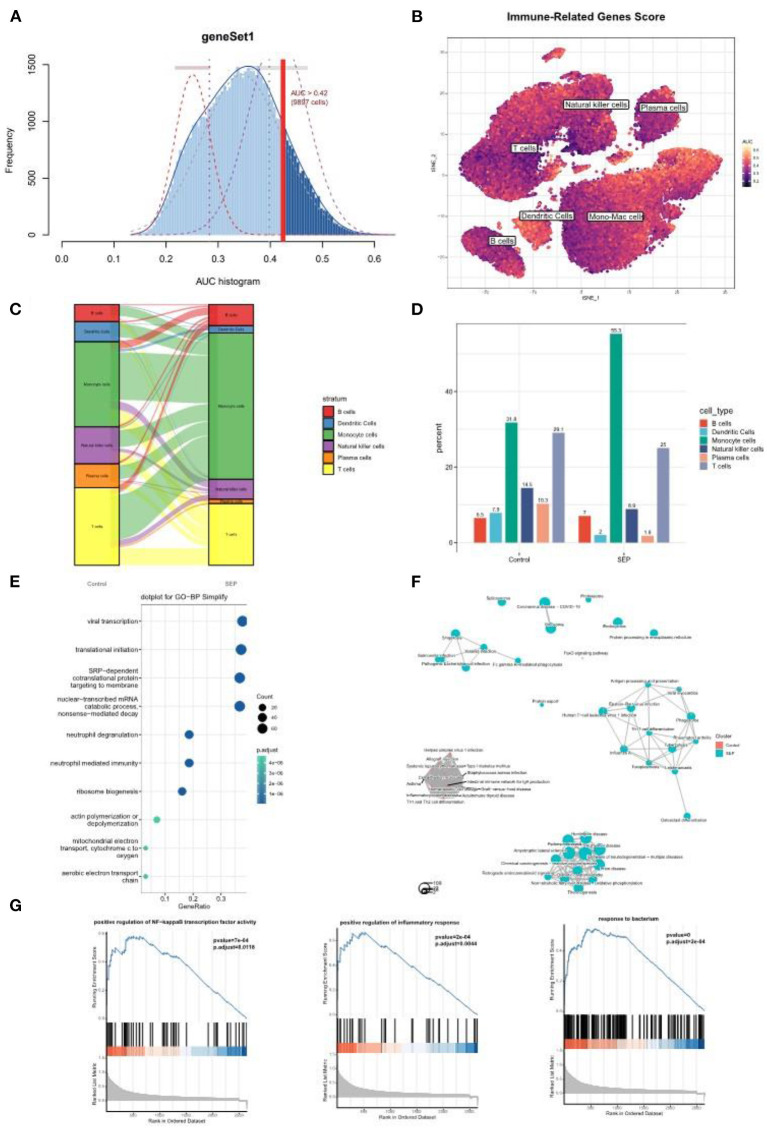
Single-cell analysis revealed a prominent monocyte/macrophage infiltration in sepsis patients. **(A)** The threshold for distinguishing gene set active cells was set at 0.42. **(B)** t-SNE plot of the immune-related genes score (IRGs score) in all of the cell types. The dendric and monocyte/macrophage cells (in yellow color) exhibited a higher IRGs Score. **(C,D)** Sankey diagram and histogram showed the proportion of monocyte/macrophage cells was significantly increased in the patients with sepsis. **(E–G)** GO and/or GSEA showed that the monocyte/macrophage clusters were mostly enriched in the proinflammatory and infection-related pathway. t-SNE, t-distributed stochastic neighbor embedding; GO, Gene Ontology; GSEA, gene set enrichment analysis.

To further investigate the role played by monocyte/macrophage clusters in the pathology of sepsis, IRG scores were first calculated using the AUCell R package. The dendric and monocyte/macrophage clusters (in yellow color) were found to exhibit higher IRG scores (Figures 6A,B), suggesting these clusters were in an active state. In addition, when compared with the healthy control group, the number of monocyte/macrophage cells was considerably elevated in the sepsis group (Figures 6C,D). It was indicated that a prominent monocyte/macrophage infiltration could be found in patients with sepsis. We then performed GO and GSEA on the monocyte/macrophage cluster. The results demonstrated that the cluster was mostly enriched in the proinflammatory and infection-related pathways, especially the NF-κB pathway, which was consistent with the functional enrichment analysis result of the Darked module in the WGCNA analysis (Figures 6E–G).

### ScRNA Profiling of Peripheral Blood Mononuclear Cells in COVID-19 Infection

**Figure 7:**
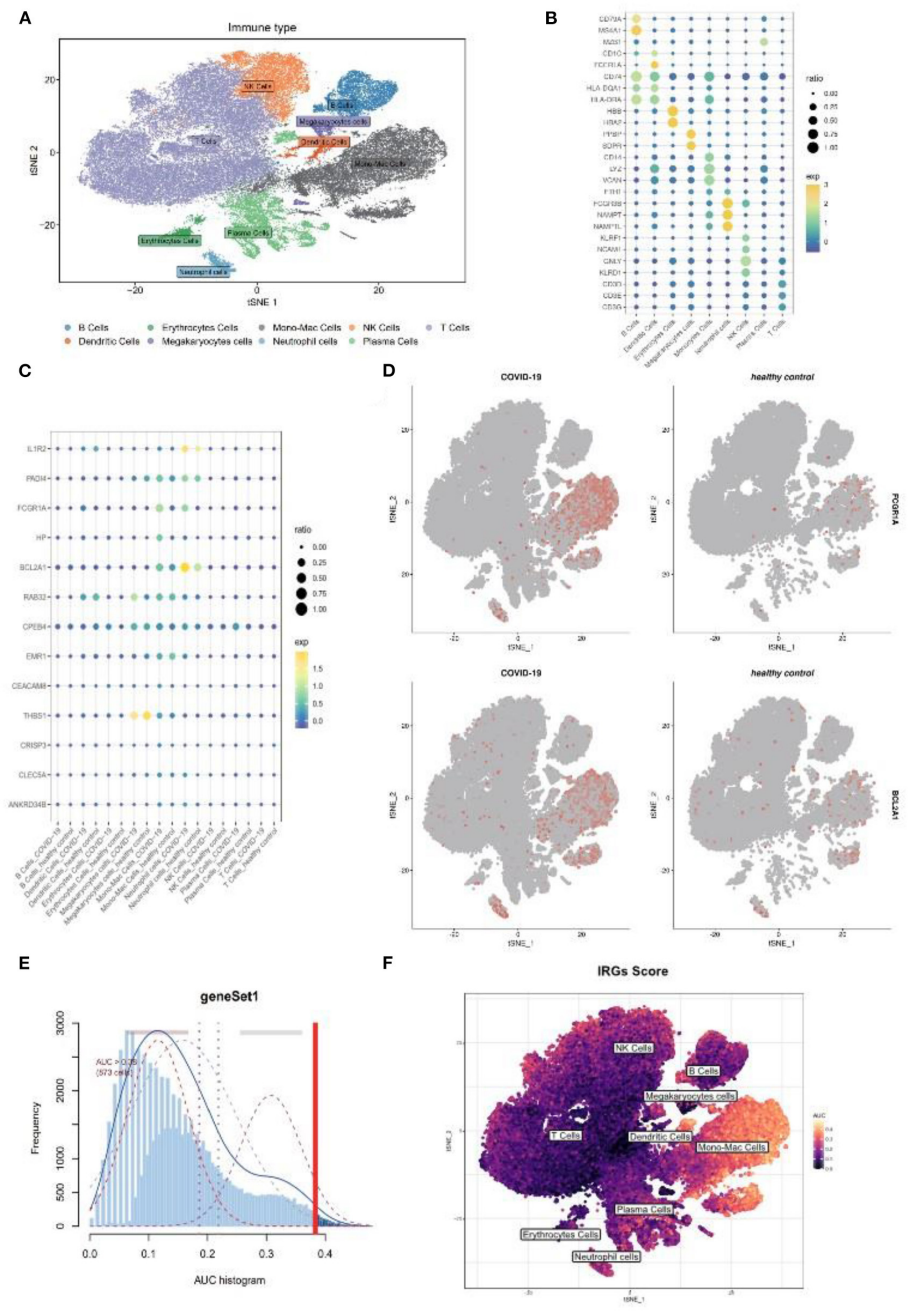
Further validation of above results in a COVID-19 scRNA dataset. **(A)** t-SNE plot visualization of nine clusters. **(B)** The respective marker genes of nine clusters in a dot plot. **(C)** Dot plot shows the expression level of target genes in the COVID-19 and healthy samples. **(D)** Expression of *FCGR1A* and *BCL2A1* using a t-SNE plot. **(E)** A threshold of 0.38 for immunity-related genes (IRGs) score calculation was selected. **(F)** t-SNE plot of IRGs score in nine clusters. t-SNE, T-distributed stochastic neighbor embedding; COVID-19, coronavirus of 2019; scRNA, single-cell RNA.

As the above scRNA dataset was focusing on bacterial infection, another virus infection scRNA dataset was also needed to verify our result. Previous studies have revealed that severe COVID-19 infection shares similar clinical symptoms and laboratory characteristics with sepsis (22). The GSE150728 dataset comprising seven in patients with COVID-19 and six healthy volunteers was selected for subsequent analysis. The clusters were annotated into nine types of cells and visualized using t-SNE analysis (Figure 7A), and their respective marker genes are shown in a dotplot (Figure 7B). The 17 genes were also examined at a single-cell level (Figure 7C). As expected, *FCGR1A* and *BCL2A1* were significantly upregulated in the disease group and were primarily expressed in the monocyte and/or macrophage cluster and the neutrophil cluster (Figure 7D). Moreover, the monocyte/macrophage cluster revealed a high IRG score (Figures 7E,F). Taken together, these results were consistent with that of bacterial sepsis in the scRNA dataset.

### Comparing the Diagnostic Performance of *BCL2A1* and *FCGR1A* as Biomarkers for Sepsis

**Figure 8:**
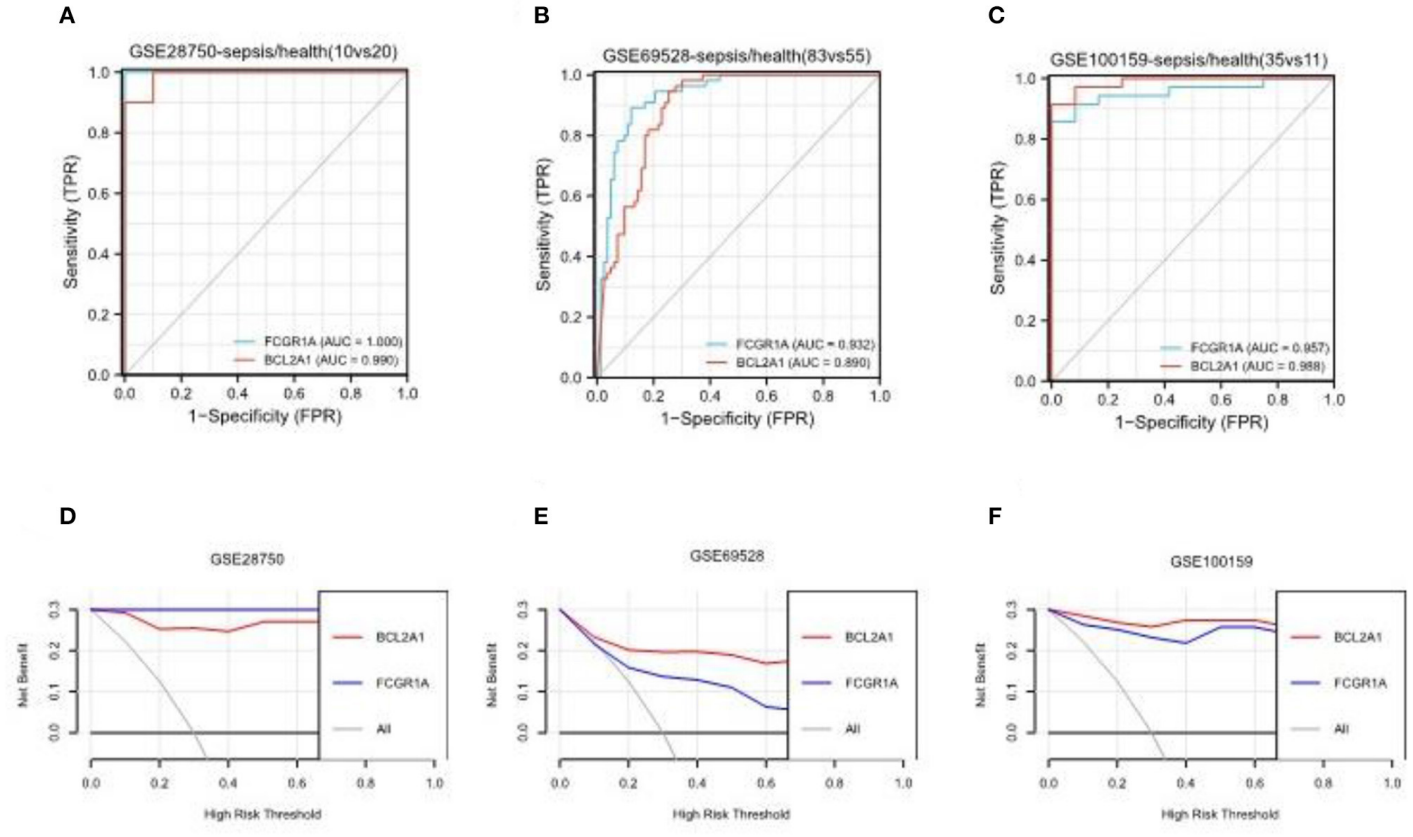
ROC curve analysis and decision curve analysis of *BCL2A1* and *FCGR1A* in three sepsis datasets. **(A–C)** *BCL2A1* revealed a high AUC value in all three datasets, suggesting its diagnostic value in sepsis. **(D–F)** DCA of *BCL2A1* and *FCGR1A* in the three datasets. ROC, receiver operating characteristic; AUC, area under the curve; DCA, decision curve analysis.

The gene *FCGR1A*, which is also called CD64, is a classic sepsis-related biomarker which has been well studied and applied in the diagnosis of sepsis (23). On the other hand, *BCL2A1* has rarely been reported in the pathogenesis of sepsis. To further verify *BCL2A1* as a novel diagnostic biomarker for patients with sepsis, we selected three other sepsis datasets (GSE28750, GSE69528, and GSE100159) to compare the diagnostic accuracy of *BCL2A1* and *FCGR1A* in the disease. The ROC curve analysis revealed that both *BCL2A1* and *FCGR1A* had a high AUC for the diagnosis of sepsis in all three datasets (Figures 8A–C). We used DCA to evaluate the clinical utility of *BCL2A1* and *FCGR1A* by qualifying the net benefit at a distinct threshold. As expected, the DCA results showed that *BCL2A1* and *FCGR1A* yielded similar clinical values in the diagnosis of sepsis. In GSE69528 and GSE100159, *BCL2A1* exhibited an even higher clinical value when compared with *FCGR1A* (Figures 8D–F).

## Discussion

Sepsis, as a prevalent and severe disease, is one of the major causes of death worldwide in the ICU (1). An early and accurate diagnosis of sepsis is crucial, as delays in prescribing appropriate therapy can greatly influence the outcome of this disease (3). Although a variety of treatment and laboratory testing technologies have been gradually applied to clinical practice, the prognosis of sepsis is still not satisfactory (24, 25). In addition, multiple sepsis-related diagnostic biomarkers have been identified, and most of them have not been clearly demonstrated as effective (26). A novel biomarker is still needed in the management of patients with sepsis. Taking the important role of monocytes and macrophages in the pathogenesis of sepsis into account, we attempted to explore sepsis-related biomarkers from the viewpoint of monocyte/macrophages. In this study, we integrated bulk-RNA sequencing data and scRNA data and identified two biomarkers (*FCGR1A* and *BCL2A1*), which were closely related to sepsis and monocyte/macrophage.

The gene *FCGR1A*, also called CD64, is a classic sepsis-related biomarker. Neutrophil CD64 was considered as a marker of neutrophil activation in acute inflammatory reaction. Multiple studies have demonstrated that neutrophil CD64 expression as a candidate biomarker for diagnosing sepsis (27, 28). Our study also found that CD64 was up-regulated in neutrophil cells at the single-cell level. In addition, it also demonstrated that CD64 was significantly elevated in monocyte/macrophage cells, which was consistent with previously published studies (29, 30). However, controversy remains about whether CD64 expression on monocytes can be a diagnostic and prognostic biomarker for sepsis (31). Some researchers found that simultaneously analyzing CD64 expression for both types of cells can improve the accuracy of diagnosis (32). It was also revealed from our study that the level of *FCGR1A* was considerably elevated in sepsis, and this gene was also correlated to the prognosis of sepsis. Nonetheless, further research is still needed to verify the results of our study in clinical practice.

The gene *BCL2A1* is a member of the B-cell lymphoma 2 (BCL2) protein family and is also an important cell death regulator. The gene exerts its antiapoptotic function by sequestering proapoptotic BCL2 proteins (33). It has been well studied in the tumor setting and has been identified as a potential target for cancer therapy (34). However, its role in the pathology of sepsis is still uncertain. Our study showed that *BCL2A1* was significantly upregulated in patients with sepsis at both the tissue- and single-cell levels. The LASSO Cox and random forest regression algorithms demonstrated that *BCL2A1* was closely related to the prognosis of sepsis.

With the development of monitoring techniques and treatment, the mortality rate of sepsis declines to 15–25%, while the in-hospital mortality rate of septic shock is still as high as 30–50% (1). *BCL2A1* was identified as a prognostic biomarker for sepsis patients in our study through LASSO and Random Forest regression analysis. However, due to the lack of demographic and clinical data, some clinical studies are needed to further analysis the efficacy of the novel marker in identification of sepsis and prognostic prediction.

In addition, to further verify *BCL2A1* as a novel biomarker for diagnosing sepsis, we selected three other sepsis datasets to perform ROC curve analysis and DCA. The results showed that *BCL2A1* had a good diagnostic value in all datasets, which indicated that it might be considered as a potential biomarker for sepsis. Most importantly, scRNA profiling showed that this gene was primarily expressed with monocyte/macrophage and neutrophil cells, which indicated that the function of *BCL2A1* was closely related to these two types of cells.

Our study also found that patients with sepsis mainly manifested a monocyte/macrophage cell infiltration. Moreover, the monocyte/macrophage cluster showed a higher IRG score, suggesting that these types of cells were in an active state during the acute stage of sepsis. The GO analysis and GSEA revealed that these types of cells were strongly correlated with inflammation activation, especially the NF-κB pathway. The activation of NF-κB can not only lead to monocyte/macrophage polarizing into an M1-like macrophage (9) but can be an important inducer of *BCL2A1* expression as well (30). This could explain partly why *BCL2A1* was significantly increased in patients with sepsis and was primarily expressed in terms of monocytes/macrophages. The effect of increasing *BCL2A1* expression on monocyte/macrophage cells appears to exert its antiapoptotic function and further exacerbate the imbalance between M1-and M2-like macrophages and eventually worsen the status of sepsis. In addition, over-expression of *BCL2A1* on neutrophils might be correlated to delayed neutrophil apoptosis (35) and can lead to immune dysfunction and persistent inflammation. Thus, further study is also needed to explore the possible mechanisms.

*BCL2A1* was identified as a promising and novel biomarker for sepsis diagnosis in our study, limitation still exists. The data of our study was downloaded from public databases, the effectiveness of clinical application is uncertain due to the lack of demographic and clinical information of patients with sepsis. Thus, further clinical studies are needed to verify the efficacy of the novel marker.

In conclusion, our study found that *BCL2A1* revealed good diagnostic and prognostic value for sepsis. The *BCL2A1* gene can be applied as a potential and novel biomarker for the management of sepsis.

## Data Availability Statement

The original contributions presented in the study are included in the article/Supplementary Materials, further inquiries can be directed to the corresponding authors.

## Ethics Statement

Ethical review and approval was not required for this study in accordance with the local legislation and institutional requirements.

## Author Contributions

JL, MZ, and Y-TY: Conception and design. L-JL: Administrative support. CZ, L-XZ, S-YY, and W-YL: Provision of study materials or patients. JL, MZ, and J-QF: Collection and assembly of data. JL, MZ, Y-TY, and S-MH: Data analysis and interpretation. All authors manuscript writing and final approval of manuscript.

## Funding

This work was supported by Shanghai Shenkang Hospital Development Center (SHDC12018106), the National Key Research and Development Program of China (2017YFC0909002) and the National Natural Science Foundation of China (81974251).

## Conflict of Interest

The authors declare that the research was conducted in the absence of any commercial or financial relationships that could be construed as a potential conflict of interest.

## Publisher's Note

All claims expressed in this article are solely those of the authors and do not necessarily represent those of their affiliated organizations, or those of the publisher, the editors and the reviewers. Any product that may be evaluated in this article, or claim that may be made by its manufacturer, is not guaranteed or endorsed by the publisher.

## References

[1] Singer M Deutschman CS Seymour CW Shankar-Hari M Annane D Bauer M. The third international consensus definitions for sepsis and septic shock (Sepsis-3). JAMA. (2016) 315:801–10. 10.1001/jama.2016.0287 26903338 PMC4968574

[2] Li H Markal A Balch JA Loftus TJ Efron PA Ozrazgat-Baslanti T. Methods for phenotyping adult patients in sepsis and septic shock: a scoping review. Crit Care Explor. (2022) 4:e0672. 10.1097/CCE.0000000000000672 35372844 PMC8970078

[3] Garnacho-Montero J Aldabo-Pallas T Garnacho-Montero C Cayuela A Jimenez R Barroso S. Timing of adequate antibiotic therapy is a greater determinant of outcome than are TNF and IL-10 polymorphisms in patients with sepsis. Crit Care. (2006) 10:R111. 10.1186/cc4995 16859504 PMC1751000

[4] Gao L Yang B Zhang H Ou Q Lin Y Zhang M. DcR3, a new biomarker for sepsis, correlates with infection severity and procalcitonin. Oncotarget. (2018) 9:10934–44. 10.18632/oncotarget.23736 29541387 PMC5834251

[5] Wu TW Tabangin M Kusano R Ma Y Ridsdale R Akinbi H. The utility of serum hepcidin as a biomarker for late-onset neonatal sepsis. J Pediatr. (2013) 162:67–71. 10.1016/j.jpeds.2012.06.010 22796049

[6] Pierrakos C Velissaris D Bisdorff M Marshall JC Vincent JL. Biomarkers of sepsis: time for a reappraisal. Crit Care. (2020) 24:287. 10.1186/s13054-020-02993-5 32503670 PMC7273821

[7] Wang T Zhang X Liu Z Yao T Zheng D Gan J. Single-cell RNA sequencing reveals the sustained immune cell dysfunction in the pathogenesis of sepsis secondary to bacterial pneumonia. Genomics. (2021) 113:1219–33. 10.1016/j.ygeno.2021.01.026 33691144 PMC7937330

[8] Fu G Deng M Neal MD Billiar TR Scott MJ. Platelet-monocyte aggregates: understanding mechanisms and functions in sepsis. Shock. (2021) 55:156–66. 10.1097/SHK.0000000000001619 32694394 PMC8008955

[9] Chen X Liu Y Gao Y Shou S Chai Y. The roles of macrophage polarization in the host immune response to sepsis. Int Immunopharmacol. (2021) 96:107791. 10.1016/j.intimp.2021.107791 34162154

[10] Davis S Meltzer PS. GEOquery: a bridge between the gene expression omnibus (GEO) and BioConductor. Bioinformatics. (2007) 23:1846–7. 10.1093/bioinformatics/btm254 17496320

[11] Kusko RL Brothers JF 2nd Tedrow J Pandit K Huleihel L Perdomo C. Integrated genomics reveals convergent transcriptomic networks underlying chronic obstructive pulmonary disease and idiopathic pulmonary fibrosis. Am J Respir Crit Care Med. (2016) 194:948–60. 10.1164/rccm.201510-2026OC 27104832 PMC5067817

[12] Langfelder P Horvath S WGCNA an R package for weighted correlation network analysis. BMC Bioinformatics. (2008) 9:559. 10.1186/1471-2105-9-559 19114008 PMC2631488

[13] Newman AM Liu CL Green MR Gentles AJ Feng W Xu Y. Robust enumeration of cell subsets from tissue expression profiles. Nat Methods. (2015) 12:453–7. 10.1038/nmeth.3337 25822800 PMC4739640

[14] Meng T Huang R Zeng Z Huang Z Yin H Jiao C. Identification of prognostic and metastatic alternative splicing signatures in kidney renal clear cell carcinoma. Front Bioeng Biotechnol. (2019) 7:270. 10.3389/fbioe.2019.00270 31681747 PMC6803439

[15] Lindeboom RG Supek F Lehner B. The rules and impact of nonsense-mediated mRNA decay in human cancers. Nat Genet. (2016) 48:1112–8. 10.1038/ng.3664 27618451 PMC5045715

[16] Reyes M Filbin MR Bhattacharyya RP Billman K Eisenhaure T Hung DT. An immune-cell signature of bacterial sepsis. Nat Med. (2020) 26:333–40. 10.1038/s41591-020-0752-4 32066974 PMC7235950

[17] Butler A Hoffman P Smibert P Papalexi E Satija R. Integrating single-cell transcriptomic data across different conditions, technologies, and species. Nat Biotechnol. (2018) 36:411–20. 10.1038/nbt.4096 29608179 PMC6700744

[18] Korsunsky I Millard N Fan J Slowikowski K Zhang F Wei K. Fast, sensitive and accurate integration of single-cell data with Harmony. Nat Methods. (2019) 16:1289–96. 10.1038/s41592-019-0619-0 31740819 PMC6884693

[19] Bhattacharya S Dunn P Thomas CG Smith B Schaefer H Chen J. ImmPort, toward repurposing of open access immunological assay data for translational and clinical research. Sci Data. (2018) 5:180015. 10.1038/sdata.2018.15 29485622 PMC5827693

[20] Aibar S Gonzalez-Blas CB Moerman T Huynh-Thu VA Imrichova H Hulselmans G. SCENIC: single-cell regulatory network inference and clustering. Nat Methods. (2017) 14:1083–86. 10.1038/nmeth.4463 28991892 PMC5937676

[21] Yu G Wang LG Han Y He QY. ClusterProfiler: an R package for comparing biological themes among gene clusters. Omics. (2012) 16:284–7. 10.1089/omi.2011.0118 22455463 PMC3339379

[22] Bost P De Sanctis F Cane S Ugel S Donadello K Castellucci M. Deciphering the state of immune silence in fatal COVID-19 patients. Nat Commun. (2021) 12:1428. 10.1038/s41467-021-21702-6 33674591 PMC7935849

[23] Hoffmann JJ. Neutrophil CD64: a diagnostic marker for infection and sepsis. Clin Chem Lab Med. (2009) 47:903–16. 10.1515/CCLM.2009.224 19642859

[24] Yu Y Zhu C Hong Y Chen L Huang Z Zhou J. Effectiveness of anisodamine for the treatment of critically ill patients with septic shock: a multicentre randomized controlled trial. Crit Care. (2021) 25:349. 10.1186/s13054-021-03774-4 34579741 PMC8474812

[25] Wang L Guo W Shen H Guo J Wen D Yu Y. Plasma microbial cell-free DNA sequencing technology for the diagnosis of sepsis in the ICU. Front Mol Biosci. (2021) 8:659390. 10.3389/fmolb.2021.659390 34124149 PMC8194294

[26] Watkins RR Bonomo RA Rello J. Managing sepsis in the era of precision medicine: challenges and opportunities. Expert Rev Anti Infect Ther. (2022) 20:871–80. 10.1080/14787210.2022.2040359 35133228

[27] Jukic T Ihan A Stubljar D. Dynamics of inflammation biomarkers C-reactive protein, leukocytes, neutrophils, and CD64 on neutrophils before and after major surgical procedures to recognize potential postoperative infection. Scand J Clin Lab Invest. (2015) 75:500–7. 10.3109/00365513.2015.1057759 26114621

[28] Yang AP Liu J Yue LH Wang HQ Yang WJ Yang GH. Neutrophil CD64 combined with PCT, CRP and WBC improves the sensitivity for the early diagnosis of neonatal sepsis. Clin Chem Lab Med. (2016) 54:345–51. 10.1515/cclm-2015-0277 26351925

[29] Hirsh M Mahamid E Bashenko Y Hirsh I Krausz MM. Overexpression of the high-affinity Fcgamma receptor (CD64) is associated with leukocyte dysfunction in sepsis. Shock. (2001) 16:102–8. 10.1097/00024382-200116020-00003 11508860

[30] Barth E Fischer G Schneider EM Wollmeyer J Georgieff M Weiss M. Differences in the expression of CD64 and mCD14 on polymorphonuclear cells and on monocytes in patients with septic shock. Cytokine. (2001) 14:299–302. 10.1006/cyto.2001.0880 11444911

[31] Hanna MOF Abdelhameed AM Abou-Elalla AA Hassan RM Kostandi I. Neutrophil and monocyte receptor expression in patients with sepsis: implications for diagnosis and prognosis of sepsis. Pathog Dis. (2019) 77:ftz055. 10.1093/femspd/ftz055 31584643

[32] Fang DH Fan CH Li J An Q Yao H Ji Q. Ratios of CD64 expressed on neutrophils, monocytes, and lymphocytes may be a novel method for diagnosis of neonatal sepsis. J Infect Dev Ctries. (2015) 9:175–81. 10.3855/jidc.4992 25699492

[33] Vogler M. BCL2A1: the underdog in the BCL2 family. Cell Death Differ. (2012) 19:67–74. 10.1038/cdd.2011.158 22075983 PMC3252829

[34] Li X Dou J You Q Jiang Z. Inhibitors of BCL2A1/Bfl-1 protein: potential stock in cancer therapy. Eur J Med Chem. (2021) 220:113539. 10.1016/j.ejmech.2021.113539 34034128

[35] Wei Y Kim J Ernits H Remick D. The septic neutrophil-friend or foe. Shock. (2021) 55:147–55. 10.1097/SHK.0000000000001620 32769816

